# Atypical Creutzfeldt-Jakob Disease Evolution after Electroconvulsive Therapy for Catatonic Depression

**DOI:** 10.1155/2011/791275

**Published:** 2011-07-03

**Authors:** Iria Grande, Juan Fortea, Ellen Gelpi, Itziar Flamarique, Marc Udina, Jordi Blanch, Raquel Sánchez-Valle

**Affiliations:** ^1^Department of Psychiatry, Institute of Neurosciences, Hospital Clinic, University of Barcelona, 08036 Barcelona, Spain; ^2^Alzheimer's Disease and Other Cognitive Disorders Unit and CJD Unit, Department of Neurology, Institute of Neurosciences, August Pi i Sunyer Biomedical Research Institute (IDIBAPS), Hospital Clinic, 08036 Barcelona, Spain; ^3^Neurological Tissue Bank, Hospital Clinic, University of Barcelona, 08036 Barcelona, Spain; ^4^Child and Adolescent Psychiatry and Psychology Department, Hospital Clinic, University of Barcelona, 08036 Barcelona, Spain

## Abstract

We describe a case report of an 80-year-old woman who presented with symptomatology compatible with an episode of major depression with catatonia. After psychiatric admission, electroconvulsive therapy (ECT) was applied, but symptoms progressed with cognitive impairment, bradykinesia, widespread stiffness, postural tremor, and gait disturbance. After compatible magnetic resonance imaging (MRI), diffusion changes, and electroencephalogram (EEG) findings the case was reoriented to Creutzfeldt-Jakob disease (CJD). The genetic study found a methionine/valine heterozygosity at codon 129 of the prion protein gene PrP^Sc^. On followup, a significant clinical recovery turned out. For this reason, EEG and MRI were repeated and confirmed the findings. The patient subsequently demonstrated progressive clinical deterioration and died 21 months later. The diagnosis was verified postmortem by neuropathology. The vCJD subtype MV2 is indeed characterized by early and prominent psychiatric symptoms and a prolonged disease duration however no frank clinical recovery has before been reported.

## 1. Introduction

Depression is a common disorder in the elderly. The prevalence of symptoms of major or minor depression in the older American population may achieve 11.9% [1]. During this period of age, depressive symptomatology may be difficult to differenciate from cognitive impairment even more since cognitive impairment progresses when depression coexists [1].

Creutzfeldt-Jakob disease (CJD), by contrast, is a rare disease characterized by prominent neurological symptoms. The term Creutzfeldt-Jakob disease (CJD) was introduced in 1922 after Hans Gerhard Creutzfeldt and Alfons Maria Jakob reported six cases of a novel neurodegenerative disease. In the actual classifications, CJD belongs to the human prion diseases and it is differentiated into the sporadic (sCJD), variant (vCJD), and familial (fCJD) subtypes [2].

We report a case of sCJD whose first diagnosis was catatonic depression due to the flourished depressive, cognitive and motor signs, with a biphasic evolution after electroconvulsive therapy (ECT), and long survival.

## 2. Case Report

**Figure 1:**
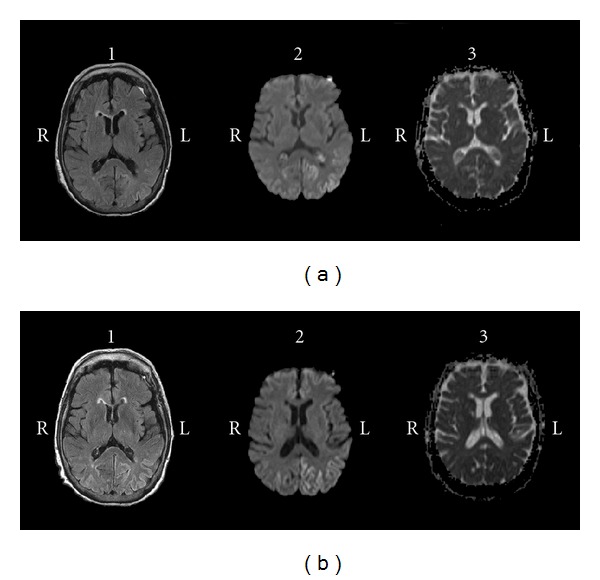
(a) Fluid attenuated inversion recovery (FLAIR), diffusion weighted imaging (DWI) and apparent diffusion coefficient (ADC) MRI demonstrate bilateral temporal-occipital cortex predominantly left hemispheric alterations with integrity of basal ganglia. (b) MRI images obtained two months later revealing the progression of the abnormalities.

An 80-year-old female was admitted to the psychiatric unit for therapeutic adjustment. She had an unremarkable personal and family medical history. Her family referred hypothymia, occasional insomnia, and anxiety after the death of her only son, when she was 78, but she had never attended a psychiatric clinic till three months before admission. During that time she lived alone and took care of herself. At the first visit, she referred anxiety, anhedonia, loneliness, fatigue, and being afraid of living alone. She was diagnosed of adjustment disorder with depressed mood. Despite treatment with different antidepressants, the patient suffered clinical deterioration. At the next psychiatric consultation apathy, loosely structured guilty, hypochondriac, and nihilistic delusions were reported. In addition, she presented reduced speech output and psychomotor retardation with pseudocatatonic postures. At that moment, she was diagnosed of a major depressive episode with catatonia and psychiatric admission for therapeutic restructuring was agreed with her family. Despite different antidepressant and antipsychotic treatments, symptoms worsened and ECT was prescribed. The patient received eight bilateral ECT sessions during the following 25 days. During this period, the patient did not present any sign of improvement. In contrast, she progressively became disoriented, inattentive, perseverative, and her speech turned to be hypophonic, monotonous, with a paucity of content and difficulties in naming and understanding complex orders. She developed rigidity, postural tremor, bradykinesia, gait disturbances, and became wheelchair bound. Due to lack of efficacy, ECT was stopped and a neurological consultation was requested. Myoclonia, ataxia, or pyramidal tract signs were not observed. Considering the differential diagnosis between CJD and epileptic status post-ECT, blood test, cerebrospinal fluid tests, as well as electroencephalogram (ECG) before and after treatment with levetiracetam, and magnetic resonance imaging (MRI) were performed. Bilateral parieto-occipital cortical hyperintensities that affected several gyri, but not basal ganglia or thalamus, were observed in diffusion-weighted imaging (DWI), MRI with diminution of the apparent diffusion coefficient (ADC), and minimal alterations in T2 fluid attenuated inversion recovery (FLAIR) sequences (Figure 1(a)). EEG showed slowness and diffuse period sharp wave complexes (PSWC) predominantly in left hemisphere that do not present without variations under antiepileptic treatment. The 14.3.3 protein assay was positive. Biochemical, hematologic alterations, viral, bacterial and parasitic infections, or tumoral processes were ruled out. At that point a diagnosis of CJD was suggested. Due to the neurologic diagnosis, the patient was transferred to the neurologic unit. There, the clinical status deteriorated till a state close to akinetic mutism with no spontaneous speech, no spontaneous movements, inattention, one-word answers to questions, perseverative movements, dysphagia, and incontinency. The family consented genetic study of the prion protein gene (*PRNP*) which detected heterozygosity for methionine/valine at codon 129 and no causative mutations. The patient was discharged to a nursing home 36 days after admission. Surprisingly, at one-month followup, the patient's neurological condition had significantly improved. She was oriented and able to maintain a simple conversation and walk by herself. Nevertheless, cognitive and depressive complains persisted and she referred lacunar amnesia of the previous 6 months. Neurological examination showed symmetric akinetic parkinsonism, reduced fluency, word-finding, difficulties, and ideomotor apraxia. The Minimental state examination score was 21. A new MRI not only confirmed the aforementioned results, but also showed an extension of the abnormalities (Figure 1(b)). In contrast, PSWC had disappeared in the EEG. Three months later, cognitive reevaluation could not be reassessed because of fatigue, and eventually the patient could not attend more to consultation due to a slowly progressive cognitive and motor impairment. She died 2 years later, 29 months after the beginning of the episode. Neuropathologic study revealed classical features of CJD with spongiform change, neuronal loss, and gliosis. Large confluent vacuoles were abundant in cortical areas and were surrounded by patchy-perivacuolar PrP^res^ deposits. In addition, frequent unicentric Kuru-type plaques in cerebellar granular layer were observed. Western-blot analysis demonstrated the presence of PrP^res^ type 2. Morphological features were compatible with the mixed MV 2K + C subtype. Concomitant brainstem Lewy-bodies were observed.

## 3. Discussion

We report a patient with atypical sCJD manifestation with psychiatric onset, and transient clinical improvement after ECT and long survival. 

Our patient presented clinically with psychiatric symptoms fulfilling criteria for major depression. While cognitive and cerebellar symptoms are the most frequent presentations of sCJD, psychiatric symptoms may also appear [3, 4], especially in the MV2 subtype [5], but these are usually associated with neurological signs and are mild in severity. Krasnianski et al. [5] reported that all MV2 subjects presented psychiatric symptoms during the course of the disease, 38% depression, although dementia and ataxia were the most common initial symptoms, and only two subjects were first diagnosed as depression. 

The long survival in this patient is consistent with the evolution of MV2 subtype. However, this case presented a biphasic evolution, and despite the fast initial deterioration, the patient survived above the median survival in published series. The transient and unexpected clinical improvement, that even generated doubts on diagnosis, is difficult to explain since there are no previous reports of such a significant amelioration. Whether concomitant brainstem predominant Lewy-body pathology might have contributed to motor disturbances or influenced the atypical clinical evolution remains a questionmark. Prolonged post-ECT delirium might have complicated the course of the disease, and thus, the ECT withdrawal accounted for the improvement, although peri-ECT amnesia persisted. Post-ECT delirium and memory loss are frequent neurological side effects of ECT. This patient had advanced age and concomitant cognitive impairment, both are risk factors for cognitive adverse effects of ECT [6]. However, Jiang et al. do not describe relevant effects of ECT in a previous case of CJD treated with ECT that suggested an especial susceptibility for post-ECT delirium in this disease [7], neither other authors [8] in short series of acute neurological conditions treated with ECT because of severe psychiatric manifestations. So other hypothesis could not be excluded. 

The atypical clinical course and low sensitivity of established tests represent a main diagnostic problem particularly in the MV2 subtype of sCJD [5]. For this reason, MRI is now considered as a key tool to evaluate the diagnosis of Creutzfeldt-Jakob disease and has been recently included in the new proposed diagnostic criteria [9]. MV2 cases usually basal ganglia and/or thalamic hyperintensities in DWI MRI, but not PSWC in EEG. However, this was not the case in our patient which presented with high signal abnormalities in cortical parieto-occipital regions either in DWI or FLAIR and PSWC at the beginning. Significant alteration in posterior areas could be related to prominent spongiform degeneration with clusters of confluent vacuoles observed in cortical areas at histology with less subcortical affection alteration. It is less probable that MRI findings were influenced by ECT as previous literature does not support signal abnormalities in DWI [10]. Concerning the transient presence of PSWC, we cannot exclude the possibility that ECT could account for this finding, as PSWC are not frequent in MV2 and disappeared after ECT withdrawal. It has been discussed that PSWC appear due to an imbalance in the subcortical, probably thalamic, pacemaker systems and the ascending reticulothalamocortical system. In our review of the literature we cannot find any report of the presence of PSWC after ECT [11]; however, mild EEG alterations can be observed post-ECT (a progressive increase in amplitudes, a slowing and greater rhythmicity of frequencies, and the development of burst patterns after repetitive ECT [12]) specially in old patients and we cannot discharge that in a predisposed brain, the ECT could generate a prolonged alteration in the ascending reticulothalamocortical system to produce PSWC. 

In conclusion, sCJD may present clinically with major depression and transient clinical recovery does not rule out CJD diagnosis. MRI is the most reliable tool in MV2 subtype diagnosis across disease course.
